# Efficacy and safety of low-dose trazodone hydrochloride for insomnia in patients with myasthenia gravis

**DOI:** 10.1186/s12883-026-04962-x

**Published:** 2026-05-08

**Authors:** Jing-ting Peng, Xiu-yun Kong, Ran Li, Yang-yue Cao, Han-qiu Jiang, Jia-wei Wang, Shi-lei Cui

**Affiliations:** Neurology Department, Beijing Tongren Hospital, Capital Medical University, Beijing, 100730 China

**Keywords:** Myasthenia gravis, Insomnia, Trazodone hydrochloride

## Abstract

**Background:**

The management of insomnia in patients with myasthenia gravis (MG) poses a clinical challenge, as MG is a well-recognized contraindication to sedative-hypnotic agents. This retrospective study aimed to assess the efficacy and safety of low-dose trazodone hydrochloride for the management of insomnia in MG patients.

**Methods:**

Clinical data were collected from MG patients with comorbid insomnia who received low-dose trazodone hydrochloride (25–100 mg, once nightly, for ≥ 4 weeks). Enrolled patients were stratified into three groups based on Self-Rating Depression Scale (SDS) scores: pure insomnia group (SDS: 0–52), mild depression group (SDS: 53–62), and moderate-to-severe depression group (SDS ≥ 63). Changes in Pittsburgh Sleep Quality Index (PSQI) scores, SDS scores, and Myasthenia Gravis Activities of Daily Living (MG-ADL) scores before and after trazodone hydrochloride administration were analyzed.

**Results:**

Total 68 MG patients were enrolled, including 17 cases of ocular MG and 51 cases of mild generalized MG. Of these patients, 18 (26.5%) were assigned to the pure insomnia group, 30 (44.1%) to the mild depression group, and 20 (29.4%) to the moderate-to-severe depression group. Post-treatment PSQI scores were significantly reduced compared to baseline across all groups (all *p* < 0.01). The mild depression group exhibited a significant decrease in SDS scores after treatment (*p* < 0.01), whereas no significant reduction in SDS scores was observed in the moderate-to-severe depression group. No patients experienced an increase in MG-ADL scores. Mild adverse events were reported in 4 patients (5.9%), including hypotension (*n* = 2), dizziness (*n* = 1), and nausea (*n* = 1).

**Conclusions:**

Low-dose trazodone hydrochloride demonstrates favorable efficacy and safety in improving insomnia in patients with stable and mild MG.

## Introduction

Myasthenia Gravis (MG) is an acquired autoimmune disease characterized by neuromuscular junction transmission impairment. Although various immunotherapies can alleviate myasthenic symptoms, a complete cure has not yet been achieved [1]. MG patients may experience psychological issues such as depression, anxiety and insomnia. Due to the differences in study participants and assessment tools, previous studies have reported the prevalence of depression and anxiety among MG patients could range from 19.6% to 64.7% and 27.8% to 45.3%, respectively. Additionally, 23% to 76.8% of MG patients have sleep disorders of varying severity [2–10]. Factors associated with comorbid insomnia in MG patients include age, anxiety, depression, disease duration, pharyngeal symptoms, respiratory distress, thymoma, the excitatory effects of glucocorticoids, and multiple nighttime medication administrations, et al. [8–10]. Chronic severe insomnia may aggravate myasthenia symptoms, prolong treatment duration and potentially precipitate myasthenic crisis [8–11].

Traditional benzodiazepines and non-benzodiazepine hypnotic agents exert sedative and muscle relaxant effects by potentiating the action of the inhibitory neurotransmitter gamma-aminobutyric acid (GABA) within the central nervous system. Owing to the risk of exacerbating MG symptoms and even inducing myasthenic crisis, such agents are generally contraindicated in MG patients [12]. However, certain antidepressants with sleep-regulating properties (such as trazodone, doxepin, mirtazapine) theoretically exhibit a lower risk of neuromuscular blockade, as they either lack anticholinergic effects entirely or demonstrate low selectivity for nicotinic cholinergic receptors in muscle tissue [12]. Thus, these agents may represent relatively safe options for MG patients with insomnia.

To date, pharmacotherapeutic research for sleep disorders in MG patients remains limited, with most existing literature consisting of case reports rather than large-sample systematic studies [11, 13, 14]. The present study aims to retrospectively evaluate the safety and efficacy of low-dose trazodone hydrochloride for the management of insomnia in MG patients.

## Methods

### Patients selection and data collection

A retrospective medical record review was performed on MG patients in the outpatient and inpatient department of our hospital’s neurology department from Jun 2018 and Jun 2025. Patients with comorbid insomnia and/or depression who received trazodone hydrochloride treatment were included.

Inclusion criteria: (1) All enrolled MG patients underwent diagnostic reconfirmation in accordance with the *Chinese Guidelines for Diagnosis and Treatment of Myasthenia Gravis (2025 Edition)* [1]. (2) The diagnosis of insomnia conformed to the *Guidelines for the Diagnosis and Treatment of Insomnia Disorder (2025 Edition)* [12]. (3) The diagnosis of depression conformed to the *Expert Consensus on the Diagnosis and Treatment of Insomnia with Depression and Anxiety in Chinese Adults* [15]. (4) Trazodone hydrochloride was administered at a dose range of 25 to 100 mg once nightly (titrated based on patient tolerability), with a minimum continuous treatment duration of ≥ 4 weeks.

Exclusion criteria: (1) Incomplete data essential for study. (2) Moderate-to-severe generalized MG with multiple interfering factors and poor compliance. (3) Concomitant use of other hypnotic agents, anti-anxiety/depression medications, or psychotropic medications either within 4 weeks before trazodone treatment or during the study period. (4) Presence of chronic conditions known to induce sleep disorders, such as chronic obstructive pulmonary disease, obstructive sleep apnea syndrome, asthma, nocturia, and chronic pain, et al. (5) Preexisting mental disorders. (6) Severe systemic illnesses, such as severe cardiovascular diseases and cardiac arrhythmias, et al. (7) A history of substance abuse or alcohol dependence.

Demographic and clinical data were extracted from inpatient and outpatient medical records, including gender, age, disease duration, Myasthenia Gravis Foundation of America (MGFA) classification, concomitant medications, duration of trazodone treatment, Pittsburgh Sleep Quality Index (PSQI), Self-Rating Depression Scale (SDS) scores and Myasthenia Gravis Activities of Daily Living (MG-ADL) scores before and after trazodone administration, and medication adverse events.

### Outcome measures

Sleep quality was assessed using the PSQI [12, 16–18]. It is a self-report questionnaire with seven components used to evaluate sleep quality over a one-month period. The score range for each component is 0 to 3, with a global score ranging from 0 to 21. A higher total score indicates poor sleep quality. Generally, a total PSQI score greater than 5 serves as the cut-off value for the diagnosis of sleep disorders [17]. The study by Liu XC et al. showed that a total PSQI score over 7 yielded a diagnostic sensitivity of 98.3% and specificity of 90.2% for sleep disorders in Chinese population [18]. In our hospital, a PSQI score > 7 is adopted as the cut-off value for diagnosing sleep disorders.

Depressive symptoms were evaluated using the SDS [12, 15, 19, 20]. The SDS is a brief self-rated scale consisting of 20 items, which is used to evaluate psychological and somatic symptoms of depression. Participants rate each item using a 4-point Likert scale (ranging from 1 to 4), based on their experiences over the past several days. The raw sum score of the SDS ranges from 20 to 80, but the results are usually presented in the form of an SDS index or a standard score, which is derived by converting the raw score to a 100-point scale. In accordance with the Chinese clinical guidelines, a standard SDS score ranging from 53 to 62 are categorized as mild depression, 63 to 72 as moderate depression, and ≥ 73 as severe depression [20]. In the present study, enrolled patients were stratified into three groups based on baseline SDS scores: pure insomnia group (SDS: 0–52), mild depression group (SDS: 53–62), and moderate-to-severe depression group (SDS ≥ 63).

The MG-ADL scale was used to assess MG-related functional status [1, 21]. It is an eight-question, clinician-directed but patient-reported questionnaire. Questions include ocular, oropharyngeal, respiratory, and extremity functions, with each response graded from 0 (normal) to 3 (most severe). Total MG-ADL score ranges from 0 to 24. Higher MD-ADL scores indicate greater functional impairment due to MG.

In accordance with the Chinese clinical guidelines, PSQI, SDS and MG-ADL scales are routine clinical examinations for the appropriate patients in our hospital. Outpatients or inpatients completed the paper-based versions of questionnaires in the hospital under the guidance of doctors. For the present study, baseline assessments of the PSQI and SDS were required to be completed within 2 weeks prior to the initiation of trazodone treatment, while follow-up assessments were mandated to be conducted at ≥ 4 weeks after treatment initiation. The observation endpoint of this retrospective study was set as the completion of the first follow-up PSQI and SDS assessments after the trazodone treatment. Changes in MG-ADL scores from baseline to post-treatment served as a core safety endpoint, reflecting alterations in disease severity. Post-treatment MG-ADL assessments were performed on an as-needed basis in response to clinical changes (e.g., exacerbation of myasthenia symptoms), and the result from worst performance was selected for data analysis.

Adverse events monitored during the study period included: (1) exacerbation of MG symptoms; (2) trazodone-related adverse reactions (e.g., hypotension, hypertension, arrhythmia, dizziness, somnolence, nausea, vomiting and allergic reactions, et al.).

### Statistical analysis

Statistical analyses were performed using SPSS 26.0. Normally distributed continuous data were presented as mean ± standard deviation. Non-normally distributed continuous data were reported as median (interquartile range, IQR [P25, P75]). Paired t-tests (for normally distributed variables) and paired Wilcoxon signed-rank tests ((for non-normally distributed variables) were utilized for pre- and post-treatment comparisons. One-way analysis of variance (for normally distributed, homoscedastic variables) and Kruskal-Wallis H tests (for non-normally distributed variables) were conducted for intergroup comparisons. Categorical data were compared using the chi-square (χ²) test. Binary Logistic regression analysis was performed for multivariate analysis. All tests were two-tailed, with statistical significance set at *p value* < 0.05.

## Results

### Baseline characteristics of patients

A total of 68 MG patients were included. Of these, 17 patients (25%) were classified as MGFA class I (ocular MG), 44 (64.7%) as MGFA class II a, and 7 (10.3%) as MGFA class II b (the latter two representing mild generalized MG). 16 cases (including 9 with MGFA II a and 7 with MGFA II b) exhibited mild bulbar palsy symptoms, presenting as mild choking, hoarseness, dysarthria, or chewing difficulty. None of the enrolled patients had respiratory muscle involvement. The main baseline data of enrolled patients, along with the PSQI, SDS, and MG-ADL scores before and after trazodone treatment, are summarized in Table 1.

**Table 1:** The baseline characteristics and scale scores before and after trazodone hydrochloride treatment in MG patients

	Total	Pure insomnia group	Mild depression group	Moderate-to-severe depression group	*p*-Value
*N*. of cases (%)	68	18 (26.5%)	30 (44.1%)	20 (29.4%)	/
Gender (Male/Female)	35/33	8/10	16/14	11/9	0.780
Age (years)	58.2 ± 14.6	55.2 ± 11.2	57.0 ± 15.3	59.3 ± 14.0	0.665
Disease course (months)	24.0 (12.0, 36.8)	21.0 (11.0, 31.0)	20.5 (8.8, 33.0)	29.5 (20.3, 42.3)	0.137
MGFA at baseline					0.296
Ⅰ	17	7 (41.2%)	7 (41.2%)	3 (17.6%)	
Ⅱa	44	8 (18.2%)	21 (47.7%)	15 (34.1%)	
Ⅱb	7	3 (42.8%)	2 (28.6%)	2 (28.6%)	
Concomitant medications					0.974
pyridostigmine bromide	67 (98.5%)	17 (94.4%)	30 (100%)	20 (100%)	
glucocorticoids	53 (77.9%)	12 (66.7%)	23 (76.7%)	18 (90.0%)	
immune-suppressants	46 (67.6%)	13 (72.2%)	19 (63.3%)	14 (70.0%)	
novel targeted drugs	14 (20.6%)	3 (16.7%)	6 (20.0%)	5 (25.0%)	
Duration of trazodone treatment (weeks)	6 (4.0, 7.8)	5.5 (4.8, 6.3)	5.5 (4.0, 8.0)	6.0 (5.0, 10.0)	0.246
PSQI scores					
pre-treatment	10 (9, 11.8)	9 (8, 11)	10 (9, 11.3)	10.5 (9, 12.8)	0.181
post-treatment	9 (7.3, 10.0)	7 (6, 8.3)	9 (8, 10)	10 (9, 10.8)	0.001
SDS scores					
pre-treatment	58 (52, 65)	45.5 (41.5, 49.25)	58 (55.8, 60)	69 (68, 71)	/
post-treatment	55 (49.3, 66.5)	44 (40, 48.5)	55 (52.8, 57.2)	70 (67.3, 71)	/
MG-ADL scores					
pre-treatment	3 (2, 5)	2 (1, 3.2)	3.5 (2, 5)	4 (3, 5)	0.053
post-treatment	3 (1, 4)	1.5 (0, 3)	3 (1, 4)	3 (2, 5)	0.019

Based on baseline SDS stratification, 18 patients (26.5%) were assigned to the pure insomnia group, 30 (44.1%) to the mild depression group, and 20 (29.4%) to the moderate-to-severe depression group. No statistically significant differences were observed among the three groups regarding gender, age, disease duration, MGFA class, concomitant medication, and trazodone treatment duration.

### Efficacy evaluation

**Fig. 1:**
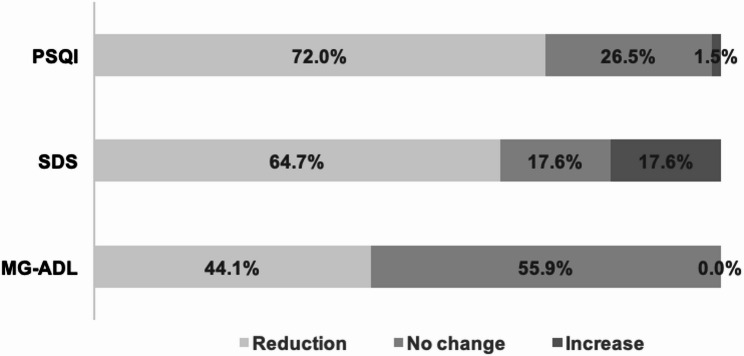
Proportion of MG patients (total 68 cases) with score changes before and after trazodone hydrochloride treatment

After trazodone administration, the PSQI and SDS scores decreased in 72% (49 cases) and 64.7% (44 cases) of MG patients, respectively. Specifically, PSQI scores remained stable in 26.5% and increased in 1.5%, while SDS scores remained stable in 17.6% and increased in 17.6% (Fig. 1). However, when compared with baseline, the overall PSQI and SDS scores were significantly reduced after trazodone treatment (all *p* < 0.001) (Table 2).

**Table 2:** Comparison of scale scores in all enrolled MG patients before and after trazodone hydrochloride treatment

	Pre-treatment	Post-treatment	Change of scores	*p*-value
PSQI score	10 (9, 11.8)	9 (7.3, 10.0)	1 (0, 2)	<0.001
PSQI range	8 ~ 15	6 ~ 13	-2 ~ 5	
SDS score	58 (52, 65)	55 (49.3, 66.5)	2 (0, 3)	<0.001
SDS range	32 ~ 72	30 ~ 74	-9 ~ 12	
MG-ADL score	3 (2, 5)	3 (1, 4)	0 (0, 1)	<0.001
MG-ADL range	0 ~ 7	0 ~ 6	0 ~ 2	

**Fig. 2:**
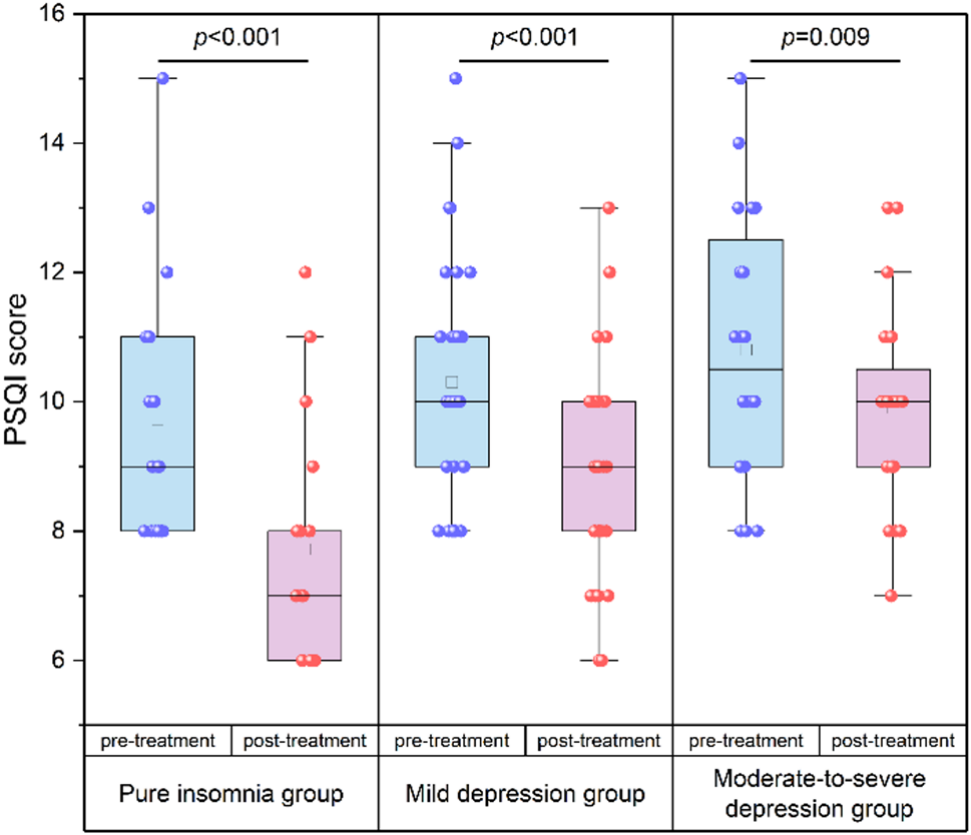
Comparison of PSQI Scores in three groups of MG patients before and after trazodone hydrochloride treatment

Post-treatment PSQI scores were significantly reduced compared to baseline across all groups (all *p* < 0.01) (Fig. 2). As seen in Table 1, the baseline PSQI scores didn’t reach statistically difference among the three groups, while the pure insomnia group demonstrated the lowest PSQI scores among the three groups after trazodone treatment (*p* < 0.001).

**Fig. 3:**
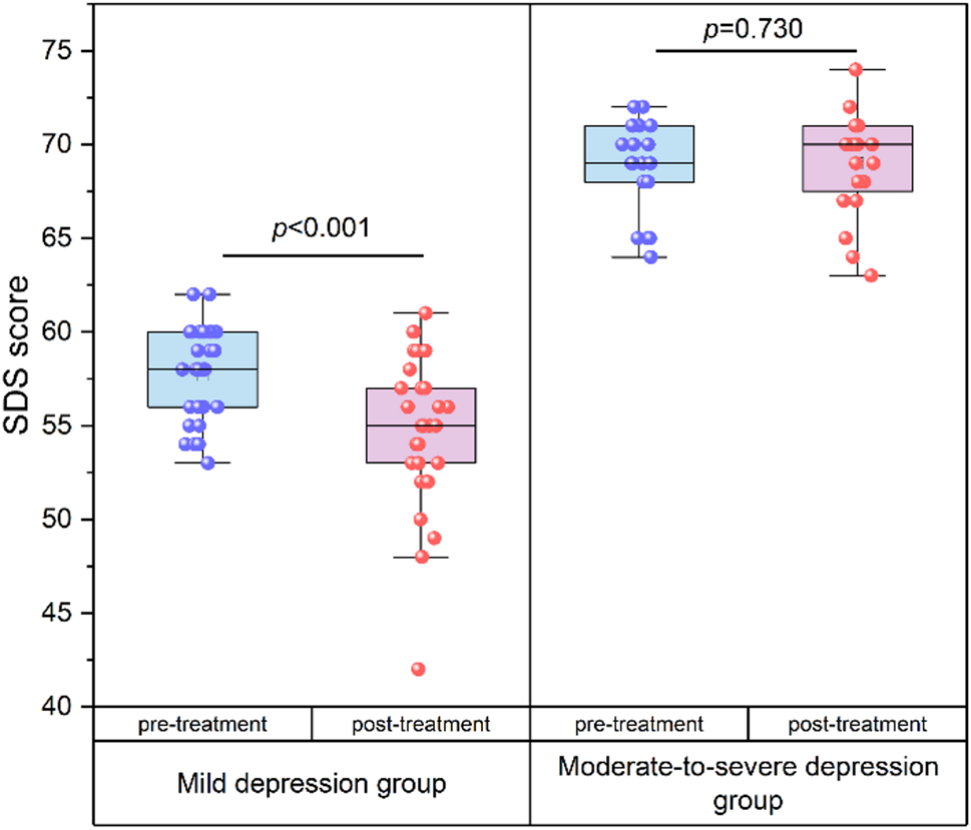
Comparison of SDS Score in MG patients before and after trazodone hydrochloride treatment

The mild depression group exhibited a significant decrease in SDS scores post-treatment (*p* < 0.001), whereas no significant change was observed in the moderate-to-severe depression group (Fig. 3). The SDS scores of the pure insomnia group did not meet the diagnostic criteria for depressive state either before or after treatment (Table 1).

To explore whether there were other factors other than trazodone treatment are associated with changes in PSQI and SDS scores, a logistic regression analysis was performed. The results indicated that group stratification was a shared influencing factor for both PSQI and SDS score improvements, while the improvements of PSQI scores and that of SDS scores were mutually associated influencing factors for each other (Table 3).

**Table 3:** Logistic regression analysis for covariate associated with improvements in PSQI, SDS, and MG-ADL scores

Dependent variable	Significant covariates*	B	*p*-value	OR (95% CI)
PSQI improvement	Group stratification	-2.640	0.040	0.071 (0.006, 0.883)
	SDS improvement	1.944	0.018	6.987 (1.399, 34.898)
SDS improvement	Group stratification	2.340	0.013	10.378 (1.630, 66.070)
	PSQI improvement	2.007	0.010	7.441 (1.613, 34.324)
MG-ADL improvement	MGFA class	-2.142	0.028	0.117 (0.017, 0.792)

*The following additional covariate were included: gender, age, disease course, MGFA class (1 = ocular MG, 0 = generalized MG), concomitant medications, trazodone treatment duration, group stratification, PSQI improvement, SDS improvement, MG-ADL improvement

### Safety analyses

Following trazodone administration, the MG-ADL score remained unchanged in 38 (55.9%) cases, and none of the cases experienced an increased MG-ADL score, myasthenia symptoms exacerbation or respiratory distress (Fig. 1). Moreover, the overall MG-ADL scores after treatment decreased significantly from baseline level (*p* < 0.001), with the pure insomnia group exhibiting the lowest MG-ADL score (*p* = 0.019) (Tables 1 and 2). In addition to pharmacological intervention, multivariate analysis revealed that MGFA class was the sole factor associated with changes in MG-ADL, that is, the improvement in MG-ADL was most significant in ocular myasthenia gravis.

A total of 4 patients (5.9%) reported adverse reactions, including 2 cases of hypotension (improved after discontinuation of antihypertensive agents), 1 case of dizziness, and 1 case of nausea. All adverse reactions were mild and did not result in treatment discontinuation.

## Discussion

Sleep disorders are highly prevalent in MG patients. In China, 39.1% to 73.1% of MG patients suffer from sleep disturbances, and approximately half experience varying degrees of emotional disorders. Notably, anxiety and depression severity can serve as predictive indicators of sleep quality in MG patients [22, 23]. Previous studies have hypothesized that MG may disrupt autonomic nervous function—via impairment of the central cholinergic system—to induce sleep disorders, though this mechanism remains controversial [24, 25]. Recent neurobiological studies have indicated that the comorbidity between internalizing mental disorders (e.g., depression, anxiety) and neurological conditions may arise from abnormalities in shared neural and cortical structural pathways [26, 27]. Current consensus identifies three main contributors to poor sleep in MG patients: (1) As a chronic, relapsing, and incurable autoimmune disease, MG imposes a heavy physical and psychological burdens on patients. (2) Pharyngeal or respiratory muscles involvement in MG can cause ventilation dysfunction and hypoxemia, and may coexist with sleep apnea syndrome or restless legs syndrome, thereby resulting in decreased sleep quality and daytime somnolence. (3) First-line MG treatments interfere with sleep via mechanisms such as enhanced gastrointestinal motility, increased salivation, frequent dosing (pyridostigmine), or excitatory / metabolic-disrupting effects (glucocorticoids) [10, 22, 23].

Many sedatives and anxiolytic-antidepressant medications are contraindicated in MG patients. Currently, the selection of medication for comorbid insomnia in MG patients is largely based on the individual experiences of clinicians. To date, there have been some case reports documenting that MG patients did not experience myasthenic symptom deterioration following benzodiazepines and antidepressant use [11, 13, 14, 28]. A prospective study in China also indicated that stable MG patients maintained disease stability after treatment with non-benzodiazepine hypnotics [16]. Given the recent regulatory approval of several novel medications with favorable safety profiles for sleep disorders, such as orexin receptor antagonists and melatonin receptor agonists, these agents are highly likely to expand treatment options for MG patients with comorbid insomnia [12].

Trazodone hydrochloride is a traditional antidepressant categorized as a serotonin antagonist and reuptake inhibitor (SARI). It has a well-defined dose-dependent profile. The effective daily dose for depression management is 100 to 150 mg or higher (maximum dose 400 to 600 mg). However, low-dose trazodone (25–150 mg/day, administered 1 h pre-sleep) exerts hypnotic effects by blocking 5-HT₂A, central α₁-adrenergic, and H₁-histaminergic receptors. Trazodone takes effect relatively quickly, usually within 1 to 2 weeks, and reaches its best effect in 2 to 4 weeks. Consequently, low-dose trazodone is recommended for insomnia treatment in Chinese clinical guidelines [12].

Theoretically, trazodone has extremely low anticholinergic activity. Nevertheless, clinicians prefer to prescribe trazodone to patients with mild and stable MG to minimize the risk of neuromuscular blockade in clinical practice. Therefore, during medical record review of this study, nearly all cases meeting the inclusion criteria were patients with ocular MG or mild generalized MG. Patients with moderate to severe MG were excluded from this study due to factors including multiple interfering factors, poor treatment compliance, and the requirement for combination therapy for sleep disorders. Additionally, considering that insomnia and depression often co-occur and influence each other, patients were further stratified by baseline SDS scores to explore whether the efficacy of trazodone for insomnia varies by depressive status.

Following trazodone treatment, the sleep quality of MG patients improved significantly, with the lowest PSQI score observed in pure insomnia group. The multivariate analysis revealed that, apart from trazodone treatment, the primary factor associated with the improvement of PSQI was the group stratification. This suggests that the improvement in sleep quality may be relatively more pronounced in MG patients with pure insomnia after low-dose trazodone administration.

Although the overall SDS score significantly decreased after trazodone treatment, the improvement in depression was mainly observed in the mild depression group. Given that the SDS incorporates assessments of sleep quality, the significant correlation between improvements in SDS scores and PSQI scores was not unexpected. Given trazodone’s dose-dependent effects (sedation predominates at low doses), the observed antidepressant effects in some patients may stem primarily from sleep-related quality- of-life improvements rather than the drug’s intrinsic antidepressant properties. The improvement in PSQI score in depression group was less than that in pure insomnia group, which also indirectly confirmed that merely treating insomnia in depression patients is insufficient.

No significant correlation was observed between the improvement of insomnia and depression with the treatment duration of trazodone. The potential explanations are the small sample size of cases and the fact that all patients’ medication duration exceeded one month, which is consistent with the theoretical time for achieving the best therapeutic effect of trazodone.

After treatment with trazodone, none of the included MG patients experienced an aggravation of myasthenic symptoms, confirming that low-dose trazodone may represent a relatively safe option for MG patients. About half of the patients showed improvement in MG-ADL score, which should be attributed to the combined effect of multi-drug therapy. Potential pharmacokinetic or pharmacodynamic interactions may exist between trazodone and MG medications treatment (such as pyridostigmine bromide, glucocorticoids, and immunosuppressants). Therefore, close monitoring of myasthenic symptoms is warranted during trazodone administration in MG patients.

In addition, it is traditionally believed that the severity of physical illness is related to the degree of psychological disorders. Recent neurobiological research has provided theoretical evidence for the mechanism by which physical functions and motor functions affect mental health outcomes through neural pathways [29]. In the present study, the MG-ADL scores of the pure insomnia group were lower than those of the other two groups of depressed patients before and after treatment, which is consistent with the theory of psychosomatic comorbidity. However, possibly due to the relatively mild disease severity and the small sample size, no significant correlation was found between the improvement of MG-ADL with the improvement of insomnia and depression, nor with the group stratification.

The α₁-adrenergic antagonism and antihistaminic activity of trazodone may still induce a spectrum of adverse reactions, with cardiovascular side effects being relatively prevalent—including hypotension, hypertension, and cardiac arrhythmias. During treatment, enhanced monitoring of the patient’s overall condition is warranted, with special attention to fluctuations in blood pressure and heart rhythm among elderly individuals.

This retrospective study has several limitations. Firstly, the sample size was small, and patients with severe generalized MG were excluded, so the research conclusion cannot be extended to the entire MG patients population. Secondly, inherent to its retrospective nature, this study conclusions were confounded by multiple factors, including the failure to collect complete clinical data (e.g., multiple objective scores reflecting disease severity, detailed regimens of concomitant medications, et al.), the lack of fixed follow-up durations and study endpoints, as well as non-uniform trazodone dosing regimens. All these factors affected further rigorous stratified analyses. Thirdly, the assessment scales employed were limited to brief and subjective scales, with no adoption of more objective and comprehensive assessment tools (e.g., multiple objective scales, complete laboratory data). Fourthly, no comparative study of multiple insomnia treatment plans was performed.

Future research should adopt prospective, large-sample, stratified designs. Additional objective assessment indicators and tools for evaluating disease severity and psychiatric comorbidity should be incorporated to enhance the validity and reliability of evaluations. Parallel-controlled designs comparing multiple interventions are also warranted to provide more reliable evidence for clinical decision-making.

## Conclusions

Insomnia and depression are common comorbidities in MG patients. However, many standard sleep aids are contraindicated in MG. This study demonstrates that low-dose trazodone exerts a beneficial effect on improve insomnia and mild depression in patients with stable mild MG, without myasthenia symptoms deterioration. Close monitoring for adverse reactions such as hypotension is warranted during treatment. Further larger-scale studies are required to clarify the safety and efficacy of trazodone in patients with moderate-to-severe MG.

## Data Availability

The datasets used and/or analysed during the current study are available from the corresponding author on reasonable request.

## Abbreviations

IQR: Interquartile Range
MG: Myasthenia Gravis
MGFA: Myasthenia Gravis Foundation of America
MG-ADL: Myasthenia Gravis Activities of Daily Living
PSQI: Pittsburgh Sleep Quality Index
SDS: Self-Rating Depression Scale
